# The Value of Petrol at Operations

**Published:** 1913-10-18

**Authors:** 


### THE VALUE OF PETROL AT OPERATIONS.

### A Suggestion by a General Practitioner.

Petrol is undoubtedly a most useful product, and one is constantly finding out some new application of it. The following description of an operation may serve to introduce yet another use for this volatile hydro-carbon. The operation was for a large fatty tumour of the right labium major in a woman of fifty.

I took the instruments from the drawer and, instead of boiling them, soaked them well in petrol. At the house the only preparation previous to our arrival consisted of the shaving. I then bathed the site of operation with petrol, and after the incision, extraction, and sewing were completed I bathed the part once again and applied dry wool. Notwithstanding the daily soiling, by the application of petrol after each micturition the wound healed by first intention. With a wound situated in such an awkward part it is impossible to keep on an orthodox dressing, and the ease of application by an unprofessional attendant made the petrol a most valuable dressing.

At my suggestion, in several of the metal works of this district small wounds are now treated at once by spraying petrol on them, and with splendid results. My idea is to render the half-destroyed cells non-poisonous by interfering with their surface tension. These small metallic burns are very difficult to heal on account not only of the dirt, but also of the enzymes which are set free from the half-burnt cells. The enzyme products poison the wound and prevent the surface of the wound taking on a healthy appearance. By means of petrol the surface tension of the enzymes is so affected as to render them quite inert, and as a consequence the wound is allowed to remain clean and unpoisoned. Under such conditions healing is much more rapid. From the patient's point of view this is of value, for as these metallic wounds are generally on the feet the man, although on compensation, is not able to get about and enjoy himself. From the employer's point of view the early return is a matter of considerable saving.

To motorists petrol is not only of value as a propelling article, but also in case of accidents it affords the best means of cleaning a wound. In cases of irregular wounds, such as scrapes or grazes, petrol affords a ready and a non-irritating means of cleansing, so that all that is required besides is cotton-wool for dressing the wound.

The non-irritating quality of petrol gives it a ready advantage over all the other antiseptics, especially those of the coal-tar group. Its one great disadvantage is that it is so inflammable; and unless operations or applications can be made away from a fire its use is contra-indicated.

